# Stable and Efficient PtRu Electrocatalysts Supported on Zn-BTC MOF Derived Microporous Carbon for Formic Acid Fuel Cells Application

**DOI:** 10.3389/fchem.2020.00367

**Published:** 2020-05-13

**Authors:** Inayat Ali Khan, Muhammad Sofian, Amin Badshah, Muhammad Abdullah Khan, Muhammad Imran, Muhammad Arif Nadeem

**Affiliations:** ^1^Chemistry of Interfaces, Luleå University of Technology, Luleå, Sweden; ^2^Catalysis and Nanomaterials Laboratory 27, Department of Chemistry, Quaid-i-Azam University, Islamabad, Pakistan; ^3^Department of Environmental Sciences, Quaid-i-Azam University, Islamabad, Pakistan; ^4^Department of Chemistry, Faculty of Sciences, King Khalid University, Abha, Saudi Arabia

**Keywords:** formic acid, carbonization, microporous carbon, cyclic voltammetry, electroxidation

## Abstract

Highly efficient, well-dispersed PtRu alloy nanoparticles supported on high surface area microporous carbon (MPC) electrocatalysts, are prepared and tested for formic acid oxidation reaction (FAOR). The MPC is obtained by controlled carbonization of a zinc-benzenetricarboxylate metal-organic framework (Zn-BTC MOF) precursor at 950°C, and PtRu (30 wt.%) nanoparticles (NPs) are prepared and deposited *via* a polyol chemical reduction method. The structural and morphological characterization of the synthesized electrocatalysts is carried out using powder X-ray diffraction (PXRD), X-ray photoelectron spectroscopy (XPS), scanning electron microscopy (SEM), transmission electron microscopy (TEM), an energy dispersive X-ray (EDX) technique, and gas adsorption analysis (BET). The FAOR performance of the catalysts is investigated through cyclic voltammetry (CV), chronoamperometry (CA), and electrochemical impedance spectroscopy (EIS). A correlation between high electrochemical surface area (ECSA) and high FAOR performance of the catalysts is observed. Among the materials employed, Pt_1_Ru_2_/MPC 950 with a high electrochemical surface area (25.3 m^2^ g^−1^) consequently showed superior activity of the FAOR (*I*_r_ = 9.50 mA cm^−2^ and *J*_m_ = 2,403 mA ${\text{mg}}_{\text{Pt}}^{-1}$) at room temperature, with improved tolerance and stability toward carbonaceous species. The superior electrochemical performance, and tolerance to CO-poisoning and long-term stability is attributed to the high surface area carbon support (1,455 m^2^ g^−1^) and high percentage loading of ruthenium (20 wt.%). The addition of Ru promotes the efficiency of electrocatalyst by offering FAOR *via* a bifunctional mechanism.

## Introduction

Direct formic acid fuel cells (DFAFCs) have gained extensive attention during the last two decades, serving as future clean energy due to their numerous benefits such as high energy density, low flux of crossover, environmental friendliness, and non-flammable properties (Chen et al., 2011; Fu et al., 2015). DFAFC is an alternative to the conventional energy conversion systems and their performance efficiency (the rate of oxidation reaction at anode) is highly dependent on platinum and platinum alloyed catalysts (Peng and Yang, 2009). DFAFCs practical realization is partly hindered by insufficient durability (due to CO-poisoning) and a high cost barrier associated with platinum, even though it has been proven that Pt is the most efficient and stable electrocatalyst (Benipal et al., 2017; Xu et al., 2017a). To decrease the cost barrier of fuel cells (FCs), one of the most noteworthy approaches is to alloy Pt with other metals such as Fe (Sun et al., 2015), Au (Li et al., 2018; Xu et al., 2018a), Ag (Lv et al., 2014), Cu (Zhang et al., 2016), and Bi (Liao et al., 2014). In FCs, the binary (PtM) catalysts have significantly decreased the cost and maintained optimum cell performance. Amongst the different PtM catalysts, PtRu combination is the most CO-tolerant in which Ru can act to oxidize CO_ads_ at a low potential, which is advantageous for the long term durability of anode catalyst (Dumont et al., 2016; Xu et al., 2017b,c).

Literature has revealed that FAOR on a PtRu-based catalyst proceeds *via* either indirect oxidation (dehydration) or direct oxidation (dehydrogenation). The indirect oxidation involves the CO formation, as an intermediate that can adsorb on the catalyst surface and can thus inhibit fuel oxidation (Park et al., 2002; Demirci, 2007), and then oxidation to CO_2_. The adsorbed CO is considered to be oxidized by Ru-OH and the poisoned Pt would regenerate. This means that FAOR relies on Pt sites, whereas Ru regenerates poisoned Pt. This is a bimetallic function which is called a bifunctional mechanism and is dependent on PtRu catalyst mutual composition (Jiang and Jiang, 2011). Jiang and Jiang (2011) have reported that the catalysts with a Pt:Ru ratio of 1:1 exhibit superior performance, in terms of the current produced, and stability in comparison to the other options. Further, Rh interconnected networks, Rh nanoneedles, and Rh nanorods were synthesized at room temperature using RhCl_3_ salt as a precursor and tartaric acid (TA), ascorbic acid (AA), hexamethylene tetraamine, and tridecylamine as capping agents. The nanostructured Rh-catalysts have demonstrated high catalytic activity toward FAOR in comparison to bulk Rh in terms of more negative onset potential and high anodic peak current density (Sathe et al., 2011; Sathe, 2013). Herein, we uncover that catalysts with a PtRu atomic ratio of 1:2 follow a direct oxidation pathway of FAOR and presented high specific, mass activities and stability.

The nature of carbon support material has an important role in the dispersion, deposition, and stability of PtRu nanocatalysts (Prado-Burguete et al., 1989; Liang et al., 2005). The use of nanostructured carbons has increased the surface area and performance of the catalysts and decreased the Pt loading (Rodriguez-Reinoso, 1998). Extensive research is being conducted on producing carbons with high surface areas and high porosity as a support for Pt loading. Du and co-workers reported the effect of N-doped graphene (NG) support on PtAu/Pt nanocrystals (Xu et al., 2018a), dandelion PtRu nanocrystals (Xu et al., 2018b), and PtAuRu nanocrystals (Wen et al., 2018) for FAOR. The PtAu/Pt NG catalyst's high mass and specific activities, vs. commercial Pt/C catalyst, was attributed to the high active surface area, synergistic and electronic interaction between the catalyst and support. However, the catalyst's long-term stability was drastically decreased to only 30–35% over 500 CV cycles (Xu et al., 2018a). There is a need to design an appropriate catalyst (PtRu) and highly conducting carbon support combination to simultaneously increase the activity and stability. In this aspect, the use of metal-organic frameworks (MOFs) as a template and precursor, for the gram scale synthesis of high surface area carbon, has attracted a lot of attention (Liu et al., 2008, 2010; Yuan et al., 2009; Hu et al., 2010; Jiang et al., 2011; Pachfule et al., 2012; Yang T. et al., 2012; Aiyappa et al., 2013; Amali et al., 2014; Yan et al., 2014; Yang et al., 2014; Khan et al., 2017). MOFs consisting of zinc metal ion and carboxylic acid or substituted imidazole ligands have been used for carbon synthesis (Yang T. et al., 2012; Aiyappa et al., 2013; Amali et al., 2014; Yan et al., 2014; Yang et al., 2014). Liu et al. (2008) reported for the first time the synthesis of porous carbon for supercapacitor application using Zn-based MOF as a template, and furfuryl alcohol as carbon precursor. A composite of polyfurfuryl alcohol and zinc-imidazolate framework-8 (ZIF-8) has also been used for the synthesis of nitrogen containing porous carbon, for H_2_ storage, and supercapacitor applications (Jiang et al., 2011; Pachfule et al., 2012). The use of furfuryl alcohol enhanced the yield and mircoporosity of the carbon.

Previously we found that high surface area carbon, synthesized by the carbonization of MOF-5, is much better than commercial carbon (Vulcan XC-72). The electrocatalyst Pt:Fe supported on porous carbon (PC-900) exhibited a current density of 450 mA cm^−2^ and a power density of 121 mW cm^−2^ during fuel cell testing, which is 4.2 times greater than that of Pt supported on Vulcan XC-72 (Khan et al., 2014). Further, we reported MOF-5-derived high-surface area carbon supported Pt-Ni and Pt-Cu (1:1) for the oxygen reduction reaction (ORR) and methanol oxidation reaction (MOR) (Khan et al., 2016a,b). The Pt-Ni (1:1) has shown a prominent positive shift of 90 mV in onset-potential while the Pt-Cu (1:1) has delivered exceptional stability and longevity in comparison to commercial Pt/C (20%). The impressive high activity and stability of the catalysts was attributed to the Pt electron interaction with transition metals and carbon support that inhibited the nanoparticles from clustering and dissolution.

Based on the above analysis, in this study we assimilated the advantages of alloy, structure, and support to successfully design microporous carbon (MPC) (Khan et al., 2015) supported PtRu alloyed nanoparticles. Remarkably, with high surface area carbon support, electron interaction of metals and *d*-band modification of Pt, the synthesized Pt_1_Ru_2_/MPC 950 presented high electrocatalytic performance toward FAOR with specific and mass activities of 9.50 mA cm^−2^ and 2,403 mA mg${}_{\text{Pt}}^{-1}$ with high durability of minimum current decay after 5,000 s. It is worth mentioning that the synthesis of microporous carbon using low-priced and self-sacrificial precursors of MOFs and its utilization in the advanced fuel cell technology, is an incredible scientific progression.

## Experimental

### Synthesis of Zn-BTC MOF

To synthesize Zn-BTC MOF, zinc acetate dihydrate [Zn (O_2_CCH_3_)_2_.2H_2_O (1.7 g, 7.76 mmol)] and 1,3,5-benzenetricarboxylic acid [BTC; C_9_H_6_O_6_ (0.21 g, 1 mmol)] were each dissolved in 20 ml dimethylformamide (DMF), separately. Triethylamine (Et_3_N, 2 drops) was added to the acid solution. The resulting solutions were mixed and kept under stirring overnight at room temperature. After reaction completion, white precipitate was obtained at folded filter paper, washed several times and finally dried in an oven at 70°C under vacuum. The purity of the product was confirmed by PXRD (Figure S1) (Huang et al., 2014; Khan et al., 2015).

### Furfuryl Alcohol/Zn-BTC MOF Mixture Preparation

Procedures of furfuryl alcohol/Zn-BTC MOF mixture preparation is already available in our previous work (Khan et al., 2015). In short, Zn-BTC MOF was mixed with 5 ml furfuryl alcohol under stirring for 12 h. After complete saturation, the mixture was collected on folded filter paper, washed several times and dried in an oven.

### Carbonization of Furfuryl Alcohol/Zn-BTC MOF Mixture

Procedures of furfuryl alcohol/Zn-BTC MOF mixture carbonization is also available in our previous work (Khan et al., 2015). In general, the mixture (1.5 g) was placed in a ceramic boat which was longitudinally located in a quartz tube, fitted in a tube furnace (Nabertherm B 180). Initially, air was evacuated by argon flow followed by heating at 150°C for 24 h to convert furfuryl alcohol to polyfurfuryl alcohol (PFA) in the Zn-BTC MOF pores. The temperature was raised to 950°C and was kept for 6 h to completely decompose the PFA/Zn-BTC MOF composite. The obtained carbon sample was labeled as: MPC 950 (microporous carbon at 950°C) and the yield was 72% (Khan et al., 2015).

### Synthesis of Catalysts

The ethylene glycol reduction method (polyol) (Chen et al., 2005; Bonesi et al., 2008) was used to synthesize the catalysts. The obtained carbon sample was sonicated in 20 ml ethylene glycol for 30 min to make a suspension, followed by heating up to 100°C with gentle stirring. For the 30% metal loadings, H_2_PtCl_6_.6H_2_O and RuCl_3_.nH_2_O were mixed in 2:1 (20% Pt and 10% Ru), 1:1 (15% Pt and 15% Ru), 1:2 (10% Pt and 20% Ru), and 0.5:2.5 (5% Pt and 25% Ru) stoichiometry, stirred for homogenous mixing and added to the carbon suspension drop wise over a time period of 30 min. The temperature of the resulting mixture was raised gradually (2°C min^−1^) to 180°C and was maintained for 4 h to complete the reaction. After reaction completion, the product was washed and dried. The synthesized catalysts were coded as Pt_2_Ru_1_/MPC 950, Pt_1_Ru_1_/MPC 950, Pt_1_Ru_2_/MPC 950, and Pt_0.5_Ru_2.5_/MPC 950. The same procedure was adopted for the Pt/MPC 950 (20%) catalyst.

### Characterization

Powder X-ray diffraction measurements were carried out using a PANalytical X-ray diffractometer (X'Pert PRO 3040/60) at a speed of 0.015 s^−1^ with Cu Kα (λ = 1.544206 Å) radiation generated at 40 kV and 30 mA. The experimental analysis of XPS and gas adsorption is available in the Supplementary Material. SEM analysis was performed using JEOL-JSM-6610LV equipped with EDX machine. TEM analysis was carried out using JEOL-JEM 2010F FE-TEM at an operating voltage of 200 kV. An elemental analysis was performed using an ICP-MS spectrometer (6100 *Sciex* Perkin Elmer) and CHNS analyzer (2400 Series II CHNS/O).

### Electrochemical Measurements

To perform the electrochemical measurements, electrode paste was prepared by sonicating 10 mg of each catalyst in 40 μL of analytical grade 2-propanol and 10 μl of Nafion 117 (binder) for 30 min to make homogenous slurry. The ink (slurry 1.2 μL of Pt_2_Ru_1_, 2 μL of Pt_1_Ru_1_, 2.5 μL of Pt_1_Ru_2_, 5 μL of Pt_0.5_Ru_2.5_ each containing 0.05 mg of Pt) was applied on the surface of a glassy carbon electrode [surface area = 0.283 cm^2^ to afford 0.18 mg cm^−2^ of Pt (Guo et al., 2005)] previously polished with alumina (0.5 μm), and the added ink was dried. The electrochemical experiments were performed using a Potentiostate/Galvanostate electrochemical workstation (Biologic SP-300). All the electrochemical measurements were carried out using three electrode systems consisting of catalyst paste coated glassy carbon (GC) as working, Ag/AgCl (3.0 mol L^−1^ KCl) as reference, and Pt wire as counter electrodes, respectively. The electrochemical active surface area (ECSA) of the catalysts was measured in H_2_SO_4_ aqueous solution (0.5 mol L^−1^). The ECSA of the catalysts was estimated from the area of the H_2_ desorption peak using equation 1;

(1) $$\begin{array}{r} \text{ECSA}={\text{Q}}_{\text{H}}/\left(210\times \text{W}\right) \end{array}$$

where “*Q*_H_” is the total charge (μC) for hydrogen desorption from only Pt active sites, as Ru is inactive in H^+^ adsorption/desorption, “*W*” represents the catalyst loading (μg) on the electrode surface, and 210 is the charge (μC) required to oxidize a monolayer of hydrogen on a bright Pt surface (Luo et al., 2013). For FAOR experiments, the electrolyte was H_2_SO_4_ (0.5 mol L^−1^) containing HCOOH (1 mol L^−1^) at room temperature. The electrolytic cell was purged with 99.9% pure Ar for 5 min before each electrochemical experiment. The CV experiments were performed at 25 mV s^−1^ potential scan rate. The CV plots of formic acid electrooxidation were normalized to specific and mass activities using the working electrode surface area, Pt loaded, and ECSA of catalysts.

## Results and Discussion

### Physical Characterizations

**Scheme 1:**
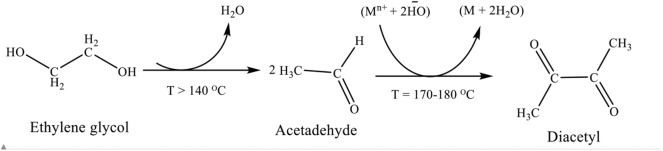
Polyol synthesis mechanism of metal nanoparticles.

Ethylene glycol reduction (polyol) method was used for the synthesis of PtRu nanoparticles. There are several reports on the reaction mechanism of the polyol synthesis method of preparing metal nanoparticles. Skrabalak et al. (2008) investigated, using a spectrophotometric method, that heating ethylene glycol in oxygen resulted to glycolaldehyde, a reductant capable of most noble metal's reduction at a high temperature. The oxidation of ethylene glycol to glycolaldehyde took place in the temperature range of 140–160°C, where the metal's reduction was prominent, when the temperature was kept at 120°C or below, no glycolaldehyde was detected in the spectrophotometric assay and the metal's reduction became diminished. The metal nanoparticles produced during the course of reaction, auto-catalyzed the ethylene glycol to glycolaldehyde. The reduction mechanism of ethylene glycol was also monitored by analyzing the volatile compounds that resulted from its oxidation (Patel et al., 2005). The formation of diacetyl was explained by the dehydration of ethylene glycol to acetaldehyde as shown in Scheme 1. During the reduction reaction the metal concertation raised to the level of saturation, where nucleation occurs, and many nuclei were formed. The nuclei rapidly grew which allowed particles development at a rate of consuming all the generated metals. Nanometric particles were generated by the envelopment of the reduced metal atoms within a suitable protective layer (LaMer, 1952; Fiévet et al., 1989).

Powder X-ray diffraction (PXRD) pattern of the synthesized Zn-BTC MOF is shown in Figure S1 (available in the Supplementary Material) which is matched with the simulated pattern reported in the literature (Huang et al., 2014; Khan et al., 2015). According to the literature reported, TGA curves in reference (Yang et al., 2018), ZnBTC MOF decomposes to ZnO-carbon at T < 600°C. The pyrolysis, under argon of the furfuryl alcohol/Zn-BTC MOF mixture at 950°C, was carried out in order to obtain pure carbon material. Furthermore, furfuryl alcohol converted to polyfurfuryl alcohol (PFA) in the channels of Zn-BTC MOF at 150°C (Bertarione et al., 2008; Radhakrishnan et al., 2011; Ullah et al., 2015) and a further rise of temperature resulted in the decomposition of PFA/Zn-BTC MOF to carbon (Bertarione et al., 2008), Scheme 2. During the carbonization process, the PFA/Zn-BTC MOF was decomposed to a ZnO-C composite at a temperature higher than 500°C. However, ZnO was reduced to elemental Zn with an increasing temperature above 900°C (Liu et al., 2010). Subsequently, metallic Zn (boiling point 908°C) vaporized away along with the Ar flow, leaving carbon only—evident in the PXRD results, Figure S2 (available in the Supplementary Material). Apart from the porosity that was generated by the vaporization of gaseous species, thermal-induced removal of zinc metal also created extra pore regions in the resulted carbon matrix. Similar to the previous reports, the XRD results of the carbon sample, PMC 950, showed two broad peaks at 2θ = 25.6° and 45° corresponding to the crystallographic (002) and (100) planes of carbon, respectively. Yang X. et al. (2012) have used polyoxyethylene bis(amine) functionalized multi-walled carbon nanotubes as a support for PtRu nanoparticles for the electroxidation of ethanol. They observed that polyoxyethylene bis(amine) acts as wrapping molecules for the well-dispersion of multi-walled carbon nanotubes which is in turn beneficial for the even distribution of PtRu nanoparticles. The idea of polymer and conducting carbon matrix integration with PtRu nanoparticles, resulted in high electrocatalytic activities and stability for the ethanol oxidation reaction.

**Scheme 2:**
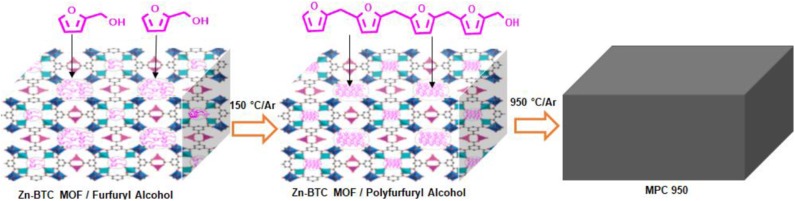
Zn-BTC MOF template infiltration with furfuryl alcohol, then polymerization of furfuryl alcohol to polyfurfuryl alcohol inside the pores of Zn-BTC MOF, followed by carbonization.

**Figure 1:**
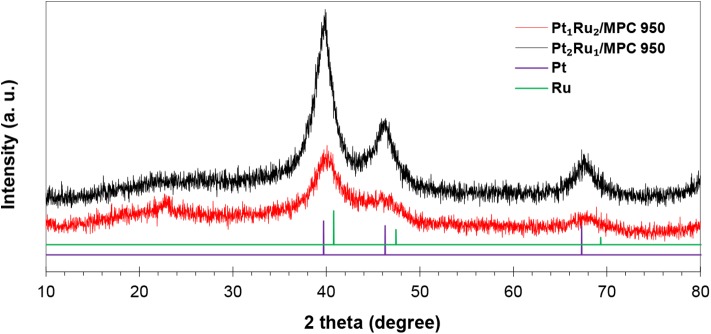
PXRD patterns of the synthesized catalysts.

The PXRD patterns of the synthesized catalysts (Figure 1 and Figure S3) have peaks at 2θ = 40.3°, 46.6°, and 67.4°, corresponding to the reflections of *fcc* Pt (JCDPS No: 00-001-1194, *Fm-3m*). In comparison to the reference PXRD pattern of Pt, the PXRD peaks of the synthesized catalysts are slightly shifted to a high 2θ value which confirmed the alloy formation. The metal atoms were excellently alloyed due to their similar atomic sizes [Pt = 0.177 nm (Clementi et al., 1967) and Ru = 0.178 nm (Clementi et al., 1967)]. The calculated crystallite size (*D*_cry_) at 2θ_111_ = 40.0° is 10.3 nm for Pt_2_Ru_1_/MPC 950, 12.5 nm for Pt_1_Ru_1_/MPC 950, and 6.4 nm for Pt_1_Ru_2_/MPC 950 catalysts, respectively. In polyol synthesis, the reducing power of ethylene glycol (EG) is highly dependent on the reaction temperature at which the aldehyde (acetyaldehyde or glycolaldehyde) molecules were generated (Patel et al., 2005; Skrabalak et al., 2008), which have a pronounced impact on the nucleation and growth kinetics of metal nanoparticles. Lobato et al. (2011) used polyol synthesis of Pt-Ru bimetallic catalysts supported on carbon nanofibers for a direct ethanol fuel cell. They observed in X-ray studies that the catalysts have a good Ru alloy level.

**Figure 2:**
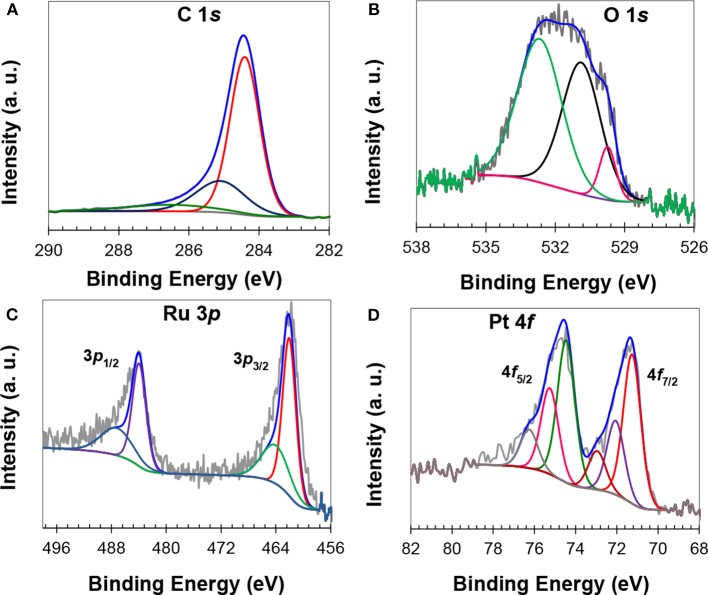
XPS core-line spectra of carbon **(A)**, oxygen **(B)**, ruthenium **(C)**, and platinum **(D)**.

XPS analysis was carried out for the electronic states' determination and composition of the catalysts. The survey scan XPS spectra of a representative catalyst, shown in Figure S4, have corresponding reflections of C, O, Pt and Ru. The curve fitting of C 1*s* spectrum resulted into three characteristic reflections at 284.4 eV due to *sp*^2^ carbon, at 285.1 eV due to *sp*^3^ carbon, and at 286.5 eV due to C–O carbon (Singh et al., 2011), respectively (Figure 2A). The XPS results showed that PFA/Zn-BTC MOF carbon is highly graphitic with ether-type surface functional groups that can enhance metal support interaction and the hydrophilicity of the catalyst. The core-line fitting of O 1*s* has resulted in three broad peaks that were assigned to the oxygen of ether carbon (532.6 eV), and oxygen bonded to metals (531 eV, and 529.7 eV), Figure 2B. The Ru 3*d* reflection of Ru^0^ occurs at 284.3 eV (Moulder et al., 1992), which is very close to the C 1*s* line spectrum of *sp*^2^ carbon; the Ru 3*p* core-line spectrum was used instead for the investigation of Ru electronic states. The high resolution XPS spectrum of Ru 3*p*, Figure 2C, showed low energy Ru 3*p*_3/2_ and high energy Ru 3*p*_1/2_ reflections. The core fitting of high energy reflection resulted in two peaks at 484.00 eV and 487.21 eV. Similarly, the low energy reflection also resulted in a doublet, one at 462.06 eV and another at 464.06 eV. The peaks at 462.06 eV and 484.00 eV were assigned to the elemental ruthenium, while peaks at 464.06 and 487.21 eV were due to the RuO_2_ (Wagner et al., 1978; Liu et al., 2004; Liang et al., 2006). The curve fittings of Pt 4*f* resulted in triplets, which were implied by the presence of elemental Pt and surface oxides (Liu et al., 2004; Liang et al., 2006; Khan et al., 2016a,b) (Figure 2D). Peaks at 71.25 eV and at 74.45 eV are due to elemental Pt, while peaks at 72.05 eV and at 75.25 eV are due to PtO. The higher binding energy peaks at 73.00 and at 76.25 eV can be assigned to PtO_2_ (Liu et al., 2004; Liang et al., 2006; Khan et al., 2016a,b). Compared to the standard value of XPS energy of Pt (71.80 eV), a negative shift of about 0.55 eV has been found for the Pt:Ru catalyst, which can be ascribed to the electron-interaction of both the metals. It has been reported in the literature that electron-interaction between the metals of Pt-alloy will result in a change of the *d*-band structure (Sarkar and Manthiram, 2010), which is significant for FAOR and catalyst stability. Moreover, it has also been reported that surface-functionalized carbon can also interact with metal nanoparticles and in turn increase the catalyst stability (Li et al., 2013). Here, a shift in binding-energy and the presence of surface ether functional groups suggested that MPC 950 electronically interact with catalyst particles and improved the stability. Siller-Ceniceros et al. (2017) reported XPS results of Pt-Ru alloyed catalysts with a shift of 0.32 eV, toward a higher binding energy of Pt^0^, in comparison to the reference value. The metal's interaction resulted in high performance of the catalyst for methanol oxidation reaction in acid medium, two times higher than that of commercial Pt/C (20%). From XPS results, the composition of a representative catalyst (Pt_1_Ru_2_/MPC 950) is 69 wt.% of carbon, 8.5 wt.% of oxygen, 16.2 wt.% of ruthenium, and 5.3 wt.% of platinum. XPS is a surface sensitive technique so that the percentage of Pt:Ru loading appeared low in comparison to the ICP-MS results. The elemental analysis of the catalyst with a Pt:Ru atomic ratio of 1:2 was also carried out through an ICP-MS and CHNS analyzer. The catalyst composed of C (69%), Pt (8.0%), and Ru (17.4%) was very close to the theoretical values of synthesis.

**Figure 3:**
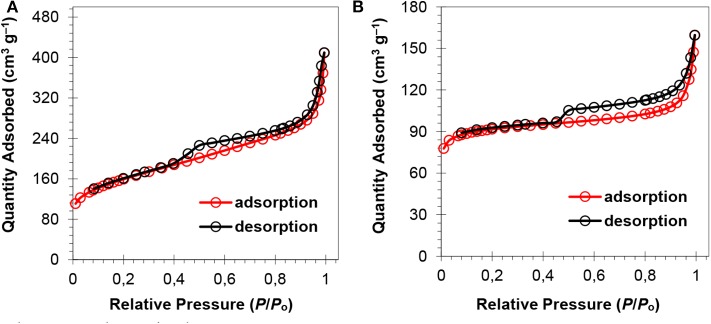
Gas adsorption isotherms of the catalysts; Pt_2_Ru_1_/MPC 950 **(A)** and Pt_1_Ru_2_/MPC 950 **(B)**.

**Figure 4:**
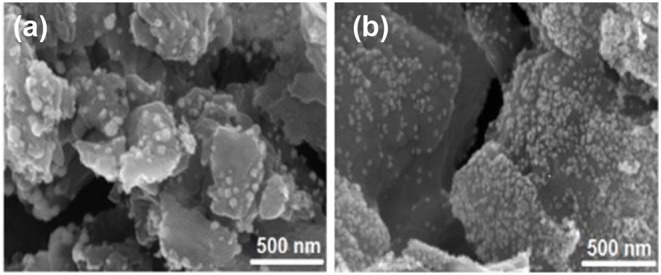
SEM images of Pt_1_Ru_2_ **(a)** and Pt_2_Ru_1_ **(b)**.

**Figure 5:**
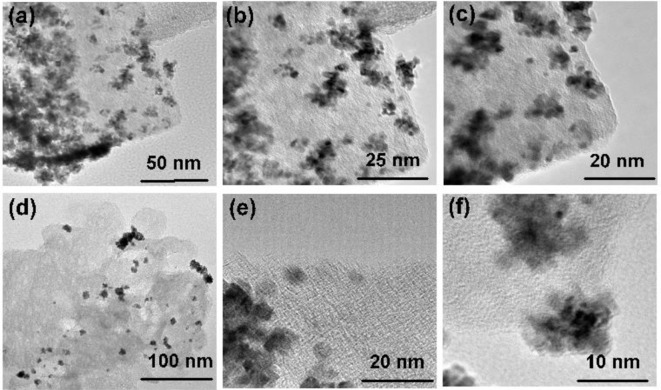
TEM images of Pt_1_Ru_2_/MPC 950 **(a–c)** and Pt_2_Ru_1_/MPC 950 **(d–f)**.

The surface area and porosity of the representative catalysts (Pt_2_Ru_1_/MPC 950 and Pt_1_Ru_2_/MPC 950) were determined using N_2_ adsorption/desorption analysis. The sorption isotherms of both the catalysts are of Type-IV with hysteresis in a high pressure region, suggesting the presence of pores from a micro to meso-range, Figures 3A,B. The BET surface areas, pore volumes, and pore sizes are given in Table 1. The high surface area and excellent porosity of the catalyst promoted interaction and mass transport of formic acid that resulted in high specific and mass activities of catalysts toward FAOR. The SEM images Figures 4A,B show irregular large flakes and porous morphology. The spherical and well-dispersed agglomerated clusters of PtRu are embedded in the carbon surface. TEM analysis of the electrocatalysts were also carried out to further investigate the surface morphology and the nanoparticles distribution on the support surface, and the obtained images are presented in Figure 5, Figure S6. It can be seen in the TEM micrographs that the particles are agglomerated/clustered-together in the form of consolidated masses which are widely distributed on the surface of the carbon (Figure 5 and Figure S6). These consolidated masses (clusters) composed of 30–50 particles with an average particle size of 6–10 nm. The PXRD, SEM, and TEM results are in close agreement.

**Table 1:** Carbons and synthesized catalyst surface parameters from the gas adsorption analysis.

**Carbons/catalysts**	**Specific surface area (m^**2**^ g^**−1**^)**	**Pore volume (cm^**3**^ g^**−1**^)**	**Pore size (nm)**	**References**
MC	1,812	2.87	–	Hu et al., 2010
MDC-1	3,174	4.06	–	Yang T. et al., 2012
PC 950	1,453	2.00	–	Khan et al., 2016a
MPC 950	1,455	2.03	–	Khan et al., 2015
CNF	231	0.45		
20% PtRu/CNF	150	0.32	–	Lobato et al., 2011
60% PtRu/CNF	97.5	0.23		
Vulcan XC72	235	0.67	–	Raghuveer and Manthiram, 2004
CMK-8-I	1,060	1.26	4.9	Maiyalagan et al., 2012
CMK-8-II	1,149	1.48	3.2	Parsons and VanderNoot, 1988
Pt_2_Ru_1_/MPC 950	532.4	0.633	4.757	This work
Pt_1_Ru_2_/MPC 950	280.0	0.246	3.528	This work

The EDX image mapping of carbon, Pt and Ru (Figure S5A) exhibited the micro-region element distribution and uniform dispersion of metal nanoparticles on support. Further, the EXD spectrum confirmed the presence of carbon, Pt, and Ru, Figure S5B. In the EDX spectrum the carbon signal is due to the carbon support (MPC 950) and the oxygen peak can be assigned to the oxygen functionalities on the surface of carbon, while the intense signal of Al is due to the aluminum foil use for sample holding during analysis.

### Electrochemical Evaluations

**Figure 6:**
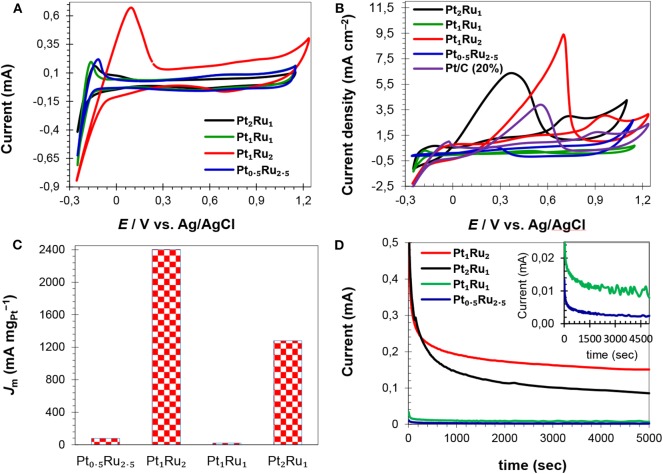
CV curves of the electrocatalysts in H_2_SO_4_ (0.5 mol L^−1^) solution **(A)**, CV curves of FAOR in H_2_SO_4_ (0.5 mol L^−1^) and HCOOH (1 mol L^−1^) mix-solution showing specific activities of the catalysts **(B)**, catalysts mass activities at their respective *E*_r_ **(C)** and current vs. time profiles of the catalysts **(D)**.

To evaluate the electrochemical performance, it is important to determine the electrochemical surface area (ECSA) of a catalyst. In general, a catalyst is more efficient in the electrochemical oxidation of small organic molecules of high ECSA and *vice versa* (Parsons and VanderNoot, 1988). Cyclic voltammetry (CV), in an acid electrolyte, is generally used to measure the ECSA. In CV curves, a platinum electrode has several redox peaks of hydrogen adsorption/desorption at negative potentials and a redox couple of platinum at positive potentials (Chen et al., 2004; Saha et al., 2008). The Pt active sites can be determined from the integrated intensity of these peaks. This means that a catalyst with high ECSA would have more active Pt sites on the surface, that would participate more actively in the electrochemical oxidation of formic acid. Figure 6A and Figures S7A–D show CV curves of the electrocatalysts for ECSA calculations. The CV curves of all the synthesized catalysts showed cathodic and anodic peaks arising from the adsorption/desorption of hydrogen (−0.25 to +0.25 V vs. Ag/AgCl) and Pt oxidation/reduction (0.40–0.80 V vs. Ag/AgCl), Figure 6A and Figure S7. For the catalyst with a Pt:Ru atomic ratio of 1:2, the adsorption and desorption peaks are intense compared to the adsorption and desorption peaks of other catalysts. Hydrogen desorption peaks (hydrogen oxidation) were used to calculate ECSA of the catalysts (Table 2) applying Equation 1. The calculated ECSA of catalysts with a Pt:Ru atomic ratio of 1:2 (25.3 m^2^ g^−1^, Table 2) is larger than the rest of the catalysts and smaller than the ECSA of the commercial Pt/C (20%; 81.5 m^2^ g^−1^) catalyst reported in our previous work (Khan et al., 2016a,b). Table 2 shows that the ECSA of PtRu catalyst with a 1:2 atomic ratio is almost double to that of PtRu catalysts with 1:1 and 0.5:2.5 atomic ratios. The high ECSA of the Pt_1_Ru_2_/MPC 950 can be attributed to the small size of the PtRu-alloy, the nano-crystalline nature, the well-dispersion on a high surface area carbon support, and the appropriate composition. Sebastián et al. (2013) have reported detailed characterization and electrochemical studies of carbon nanofibers supported PtRu nanoparticles for methanol and ethanol. A micro-emulsion procedure was adopted for the deposition of metal nanoparticles. Despite the relatively low surface area of the carbon nanofibers PtRu nanoparticles up to 2 nm in size, with a good distribution on the surface (as confirm by TEM), and high electrochemical surface areas (110–140 m^2^ g^−1^; determined by CO striping) were obtained. Schwarz et al. (2015) explored Pt(111) surface electrochemistry for FAOR and they found, using DFT calculations, that high coverages of Pt sites by formate anion leads to a large reaction barrier, which limits the availability of active sites, and a decrease in the reaction rate. Here, the formic acid oxidation reaction was studied in acidic medium in order to prevent formate ion formation, which would lead to high coverages of adsorbates on the catalyst surface and a low reaction rate.

**Table 2:** Electrochemical parameters of the reported and synthesized electrocatalysts.

**Catalysts**	**ECSA (m^**2**^ g^**−1**^)**	**^a^*E*_**f**_ (V)**	**^b^*E*_**r**_ (V)**	**^c^*I*_**r**_ (mA cm^**−2**^)**	**^d^*I*_**f**_/*I*_**r**_**	**^e^*J*_**m**_ (mA ${\text{mg}}_{\text{Pt}}^{-1}$)**	**References**
PtAgCu@PtCu	19.0	–	–	1.52	–	–	Fu et al., 2015
Pt_79_Fe_21_/NG	–	–	–	–	–	186	Sun et al., 2015
PtAu-on-Au	4.3	–	–	–	2.28	228	Li et al., 2018
Pt_1_Au_1_/NG	12.35			14.95		1,847.1	Xu et al., 2018a
PtBi NPL/XC 72	–	–	–	–	–	1,800	Liao et al., 2014
Pt_38_Ru_62_/PS-MWCNTs	32	0.95	0.76	202.8	1.63	–	Jiang and Jiang, 2011
Pt_1_Ru_1_/NG	10.15	–	–	18.30	–	1,857.4	Xu et al., 2018b
Pt_1_Au_1_Ru_1_/NG	7.20	–	–	14.50		1,044.1	Wen et al., 2018
PtCu(1:1.2)/RGO	42.6	–	–	2.08	0.87	–	Li et al., 2013
PtRu	80/CO*	–	–	0.080 at 20°C	–	–	Jiang and Kucernak, 2009
Pt/Ru/Pt	–	–	–	346	–	–	Lemos et al., 2007
PtAu/graphene	–	–	–	–	–	2,310	Zhang et al., 2011
Pt_2_Ru_1_/MPC 950	20.0	0.73	0.40	6.40	0.48	1,280	This work
Pt_1_Ru_1_/MPC 950	12.0	0.79	0.50	0.28	–	22.6	This work
Pt_1_Ru_2_/MPC 950	25.3	0.97	0.70	9.50	0.33	2403	This work
Pt_0.5_Ru_2.5_/MPC 950	13.2	–	–	0.60	–	79.3	This work

a *E_f_, forward peak potential*.

b *E_r_, reverse peak potential*.

c *I_f_, forward peak current density*.

d *I_r_, reverse peak current density*.

e *J_m_, mass current density*.

* *The ECSA was measured using CO-stripping method*.

The CV curves of the synthesized and commercial catalysts, run in the H_2_SO_4_ (0.5 mol L^−1^) and HCOOH (1 mol L^−1^) mixture are shown in Figure 6B and Figure S7. The catalysts Pt_2_Ru_1_/MPC 950 and Pt_1_Ru_2_/MPC 950 presented almost similar features of FAOR (Figures S7A,C), whilst Pt_1_Ru_1_/MPC 950 and Pt_0.5_Ru_2.5_/MPC 950 have shown different behaviors (Figures S7B,D). In the CV curves of Pt_2_Ru_1_/MPC 950 and Pt_1_Ru_2_/MPC 950, a shoulder (at about 0.30 V to 0.5 V) and a broad oxidation peak (in the range of 0.60 V to 1.0 V) in the anodic scan are ascribed to the oxidation of formic acid *via* the dehydration pathway. The oxidation of formic acid on the surface of the catalysts during the positive scan of potential, led to the formation of CO that blocked Pt-sites and inhibited further HCOOH oxidation (Lemos et al., 2007; Jiang and Kucernak, 2009). In the cathodic scan, there are peaks at 0.38 V in the CV curve of Pt_2_Ru_1_/MPC 950 (Figure S7A) and at 0.70 V in the CV curve of Pt_1_Ru_2_/MPC 950 (Figure S7C) that can be assigned to the oxidation of formic acid *via* the dehydrogenation pathway. In the cathodic scan, no other peaks were observed, which implied minimum CO-poisoning and good catalytic activity (Lemos et al., 2007; Schwarz et al., 2015). In the case of Pt_1_Ru_1_/MPC 950 an anodic peak at 0.80 V and a cathodic peak at 0.50 V were observed, while in the case of Pt_0.5_Ru_2.5_/MPC 950 an anodic peak at 0.70 V and a cathodic peak at 0.28 V were observed, Figures S7B,D. These peaks arise due to the dehydration reaction of formic acid (Jiang and Jiang, 2011; Zhang et al., 2011). The CV profiles revealed that catalysts that have a PtRu atomic ratio of 2:1 and 1:2 proceeded FAOR *via* the dehydrogenation reaction, while catalyst that have a PtRu atomic ratio of 1:1 and 0.5:2.5 proceeded FAOR *via* the dehydration reaction. The forward peak (*E*_f_) of Pt_1_Ru_2_ is at 0.97 V, while that of Pt_2_Ru_1_ and Pt_1_Ru_1_ is at 0.73 V and 0.79 V, respectively (Table 2). As compared to Pt_1_Ru_2_, the *E*_f_ of Pt_2_Ru_1_ and Pt_1_Ru_1_ is shifted negatively but their *J*_m_ (based on *E*_r_) is comparatively low (Table 2). The ratio of forward to reverse peak current densities is a parameter that judges the CO-tolerance of a catalyst. The smaller the ratio, the lower the tolerance of a catalyst in FAOR. Herein, the observed values of *I*_f_/*I*_r_ are given in Table 2 and a 0.33 value was found for the Pt_1_Ru_2_/MPC 950 catalyst, presenting good CO-tolerance. The synthesized catalysts can be arranged in a decreasing order of CO-tolerance as; Pt_1_Ru_2_ > Pt_2_Ru_1_ > Pt_0.5_Ru_2.5_ > Pt_1_Ru_1_.

The catalysts specific and mass activities of FAOR are shown in Figure 6C and Figures S8A,B, and the data are given in Table 2. The catalysts in terms of reverse peak current density (*I*_r_) and mass current per ECSA of catalyst (*J*_m_) were found to be; Pt_1_Ru_2_/MPC 950 (9.50 mA cm^−2^ and 2,403 mA mg${}_{\text{Pt}}^{-1}$) > Pt_2_Ru_1_/MPC 950 (6.40 mA cm^−2^ and 1,280 mA mg${}_{\text{Pt}}^{-1}$) > Pt_0.5_Ru_2.5_/MPC 950 (0.60 mA cm^−2^ and 79.3 mA mg${}_{\text{Pt}}^{-1}$) > Pt_1_Ru_1_/MPC 950 (0.28 mA cm^−2^ and 22.6 mA mg${}_{\text{Pt}}^{-1}$). The high *I*_r_ and *J*_m_ values of the Pt_1_Ru_2_ catalyst for FAOR can be attributed to its high specific surface area, high porosity, and high pore volume, which ensures enhanced metal dispersion and high mass transportation during electrocatalysis. A cross-analysis of the carbon support, ECSA, and *J*_m_ of the alcohol oxidation reactions have previously been reported (Sebastián et al., 2013). Different electrochemical performances were found for methanol and ethanol using carbon nanofibers of varying graphitic nature. Methanol oxidation reaction is favored using highly crystalline (highly graphitic) carbon nanofibers of low porosity, while ethanol oxidation is favored by low graphitic carbon nanofibers of high porosity. Functionalized carbon nanotubes were used as a support of PtRu nanoparticles for methanol oxidation reaction (Cheng et al., 2014). Polyethylenimine and 1-aminopyrene functionalized carbon nanotubes supported PtRu nanoparticles showed extremely high electrocatalytic activity and stability toward methanol oxidation reaction as compared to the tetrahydrofuran functionalized carbon nanotubes supported PtRu nanoparticles. The superior activities of the catalysts on functionalized carbon nanotubes were attributed to the interaction of the nitrogen-containing functional groups of polyethylenimine and 1-aminopyrene and PtRu nanoparticles assembled on carbon nanotubes. The effect of carbon support and Ru was further investigated by comparing the electrochemical results of Pt_1_Ru_2_/MPC 950, Pt/MPC 950 (20%), and commercial Pt/C (20%), as shown in Figure S9. The MPC 950 carbon supported Pt (20%) catalyst showed lower current density than the commercial carbon supported Pt (20%) catalyst. The low current density of Pt/MPC 950 is probably due to high CO-poisoning of Pt in the absence of Ru.

The catalysts electrochemical stability was investigated through chronoamperometry (CA) experiments which were carried out for 5,000 s in N_2_-saturated H_2_SO_4_ (0.5 mol L^−1^) and HCOOH (1 mol L^−1^) solution at 0.4 V vs. Ag/AgCl, followed by CV cycles at 25 mV s^−1^. The current-time response of the catalysts is given in Figure 6D. All the synthesized catalysts have exhibited a rapid potential-static current decrease in the initial stage and a pseudo-steady state after ~700 s, and then a very slow current-decay with time. A fast-initial decrease of the current is due to the adsorption of the bisulfate/sulfate anion (from sulfuric acid) (Kolics and Wieckowski, 2001) and poisonous carbonaceous intermediates (generated in the oxidation reaction) on the catalyst surface that decreased the electrocatalytic performance. Among the catalysts, PtRu with an atomic ratio of 1:2 exhibited a low initial current decrease and a relatively high current over the 5,000 s, indicating long term stability and high CO-tolerance. The catalyst of PtRu with an atomic ratio of 2:1 also maintained a high current over the entire period of test-time in comparison to the other two options. However, the catalysts PtRu with an atomic ratio of 1:1 and 0.5:2.5 have a very low current after 5,000 s, which means lower stability and high CO-poisoning. The CA results suggested that the order of the catalysts stability after 5,000 s as; Pt_1_Ru_2_ > Pt_2_Ru_1_ > Pt_1_Ru_1_ > Pt_0.5_Ru_2.5_.

**Figure 7:**
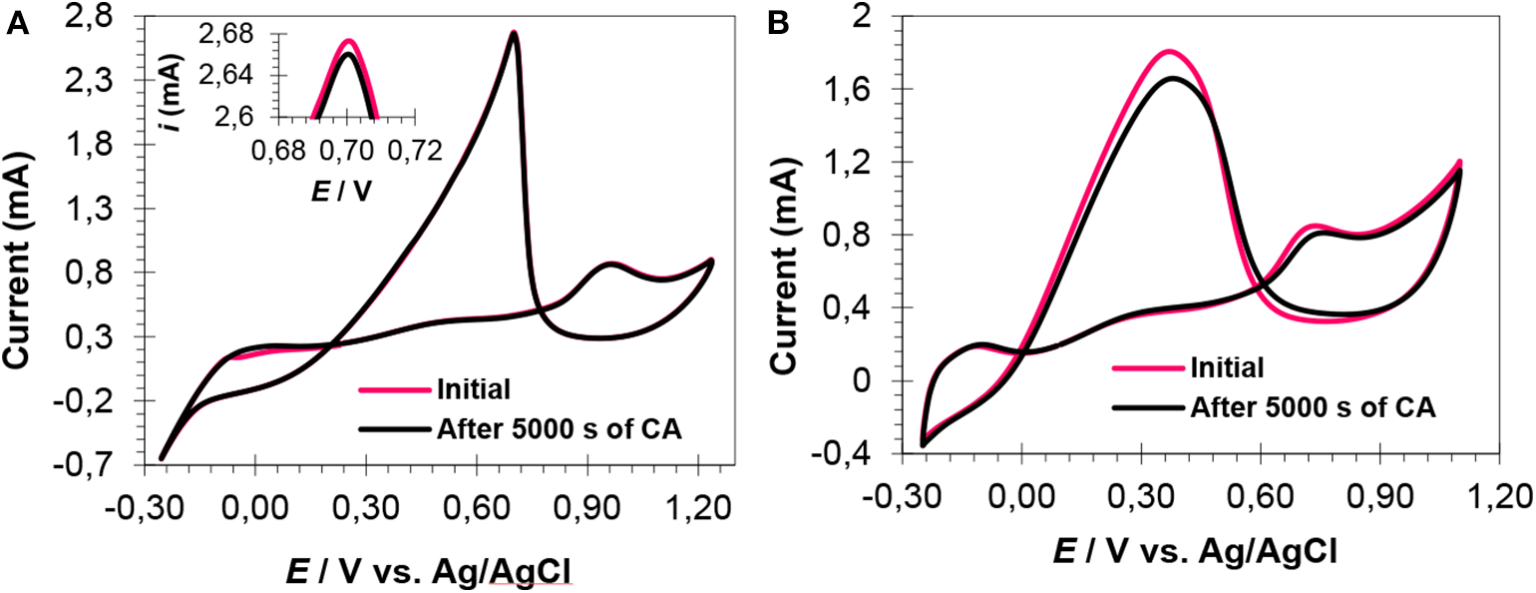
CV curves (after CA experiments) of Pt_1_Ru_2_/MPC 950 **(A)** and Pt_2_Ru_1_/MPC 950 **(B)** electrocatalysts in H_2_SO_4_ (0.5 mol L^−1^) and HCOOH (1 mol L^−1^) mix-solution.

CV cycles, after 5,000 s, were run to check the response of catalysts toward FAOR and the results are shown in Figure 7. A slight decrease in reverse peak current was observed after 5,000 s for the PtRu catalyst with an atomic ration of 1:2, Figure 7A (inset), showing the catalyst's excellent stability and high CO-tolerance. The CV curves of the PtRu catalyst with an atomic ratio of 2:1, showed a pronounced decrease in current (from 1.80 to 1.65 mA), Figure 7B. The decrease in current of the catalyst can be attributed to the agglomerated large particles (as evident from TEM analysis), high percent of Pt loading, and high CO-poisoning.

**Figure 8:**
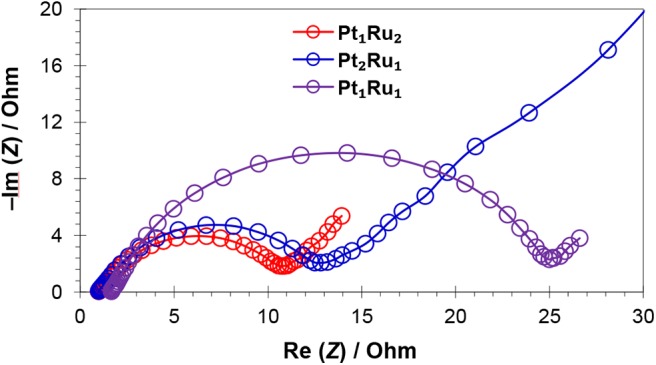
Nyquist plots of MPC 950 supported catalysts in H_2_SO_4_ (0.5 mol L^−1^) and HCOOH (1 mol L^−1^) solution.

Electrochemical impedance spectroscopy (EIS) was carried out to investigate the catalyst conductivity and frequency response, Figure 8 and Figure S10. The EIS measurements were carried out in the frequency range of 10 mHz to 10,000 Hz using a H_2_SO_4_/HCOOH (0.5/1 mol L^−1^) solution. The Nyquist plots at 0.35 V (close to *E*_r_) vs. Ag/AgCl for the three catalysts are presented in Figure 8 and Figure S10. Each spectrum consists of a semicircle, the intercept of which at the real axis in their low frequency region, shows the total resistance and ~45° slope, which can be attributed to the capacitance of catalysts. The value of the intercept, with the real axis in the high frequency region, gives the electrolyte resistance (*R*_elec_), while the diameter of the semicircle gives charge transfer resistance (*R*_ct_) (Wen et al., 2018; Xu et al., 2018b). The straight line on the imaginary part represents the double layer capacitance (*C*_dl_), especially in case of Pt_2_Ru_1_/MPC 950, which is developed between the electrode surface and ions generated after electrochemical oxidation of formic acid. The *R*_elec_ remained the same, at about 0.25 Ohm for the catalysts, showing a similar concentration and ionic strength of the electrolyte solution for every electrochemical experiment. The “*R*_ct_” values are 10.7, 13.5, and 25.2 Ohm for Pt_1_Ru_2_/MPC 950, Pt_2_Ru_1_/MPC 950, and Pt_1_Ru_1_/MPC 950 electrocatalysts, respectively. Generally, the “*R*_ct_” decreases with increasing electrical conductivity, which is obvious for Pt_1_Ru_2_/MPC 950 and Pt_2_Ru_1_/PC 950 catalysts from the Nyquist plot that was implied on their superior electrocatalytic activities toward formic acid oxidation. Furthermore, very high resistivity has been shown by PtRu with and atomic ratio of 0.5:2.5, Figure S10.

## Conclusions

PtRu crystalline nanoparticles were deposited on Zn-BTC MOF derived microporous carbon material *via* a polyol reduction method and the resulting catalysts were used as electrode materials for formic acid oxidation reaction (FAOR). The X-ray analysis confirmed the alloyed nature of Pt-Ru and the *d*-band structure modification of Pt. The microscopic analysis revealed random distribution of the nano-crystalline particles (5 to 10 nm) on carbon support. The Pt_1_Ru_2_/MPC950 electrocatalyst demonstrated excellent electrochemical performance in terms of ECSA (ca. 25.3 m^2^ g^−1^), FAOR specific and mass activities (9.50 mA cm^−2^ and 2,403 mA mg${}_{\text{Pt}}^{-1}$). In chronoamperometry experiments, the Pt_1_Ru_2_/MPC950 catalyst demonstrated high current-stability over 5,000 s and a minimum current-lose as observed in the immediate CV experiment. The high catalytic activity and excellent stability of the electrocatalyst was attributed to the alloy formation, nano-crystalline structure, high surface area carbon support, and appropriate composition. The MOFs derived high surface area carbon materials, for direct formic acid fuel cells catalysis, can be considered as an alternative to conventional carbon and is the key finding of this work.

## Data Availability Statement

All datasets generated for this study are included in the article/Supplementary Material.

## Author Contributions

All authors listed have made a substantial, direct and intellectual contribution to the work, and approved it for publication.

## Conflict of Interest

The authors declare that the research was conducted in the absence of any commercial or financial relationships that could be construed as a potential conflict of interest.
