# Supplementary material for: Stable and Efficient PtRu Electrocatalysts Supported on Zn-BTC MOF Derived Microporous Carbon for Formic Acid Fuel Cells Application
Source: Front Chem. 2020 May 13;8:367. doi: 10.3389/fchem.2020.00367 (PMC7237749; doi:10.3389/fchem.2020.00367)
Supplement: Supplementary file 1 [file Data_Sheet_1.docx]

**SUPPORTING INFORMATION**

**Stable and Efficient PtRu Electrocatalysts Supported on Zn-BTC MOF Derived Microporous Carbon for Formic Acid Fuel Cells Application**

Inayat Ali Khan,^a,b^* Muhammad Sofian,^b^ Amin Badshah,^b^ Muhammad Arif Nadeem,^b^*

^a^*Chemistry of Interfaces, Luleå University of Technology, SE-97187 Luleå, Sweden*

^b^*Catalysis and Nanomaterials Lab 27, Department of Chemistry, Quaid-i-Azam University, Islamabad 45320, Pakistan*

*Corresponding:

Email: [inayat.khan@ltu.se](mailto:inayat.khan@ltu.sek), Phone: +46 (0) 920491738 (Dr. I.A. Khan)

Email: [manadeem@qau.edu.pk](mailto:manadeem@qau.edu.pk), Phone: 092 051 90642062 (Dr. M.A. Nadeem)

**Characterization**

**XPS**: For XPS surface analysis Kratos AXIS Ultra DLD instrument with background vacuum better than 2×10^–9^ mbar was used. The sample spot size were of 500 µm, Al K_α_ (energy *hν* = 1486.68 eV) was the radiation source and power of 164 W (10.8 mA and 15.2 kV) was used for each analysis. The adventitious carbon C 1*s* peak position were used as a reference.

**Gas adsorption analysis**: N_2_ adsorption/desorption measurements were carried out using the Accelerated Surface Area & Porosimetry System 2020 supplied by Micromeritics Instruments Inc. Catalyst samples Pt_2_Ru_1_/MPC 950 (46 mg) and Pt_1_Ru_2_/MPC 950 (29 mg) were loaded separately into a glass analysis tube and outgassed for 3 h under vacuum at 200 °C prior to measurement. The isotherm was measured at 77 K and data was analyzed using Bruner-Emmett-Teller (BET) model to determine the surface area and porosity was calculated using MicroActive software *v*3.00.

**Fig. S1** PXRD patterns of Zn-BTC MOF.


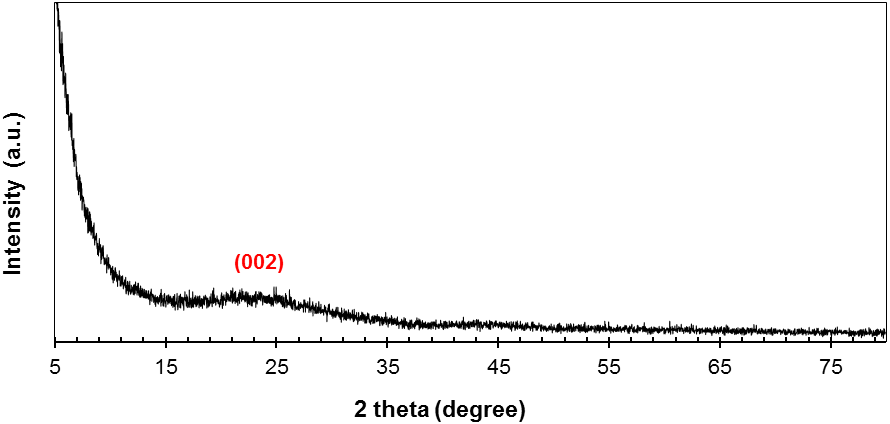

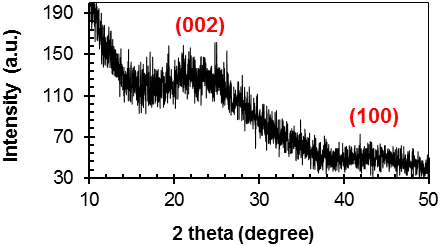


**Fig. S2** PXRD pattern of the synthesized carbon (MPC 950).

**Fig. S3** PXRD patterns of the synthesized catalysts.


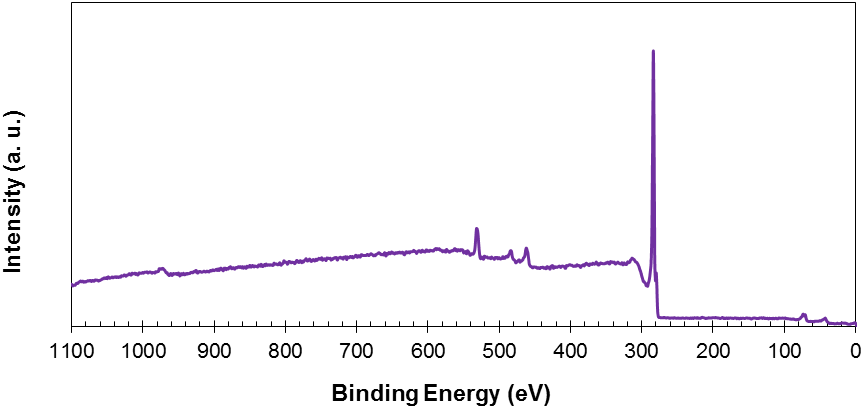


Pt

C

Ru

O

**Fig. S4** XPS survey spectra of the representative catalyst; Pt_1_Ru_2_/MPC 950.


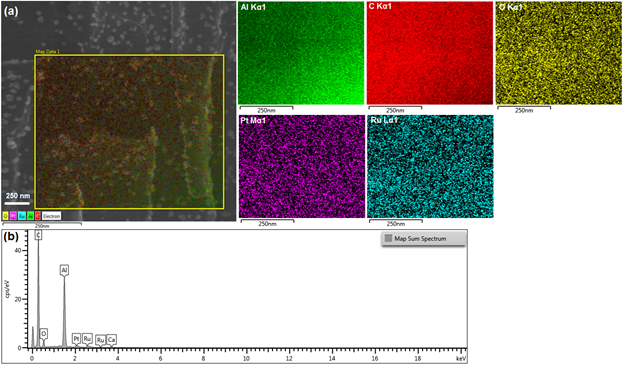


**Fig. S5** EDX elemental mapping (a) and spectrum (b) of a representative catalyst.


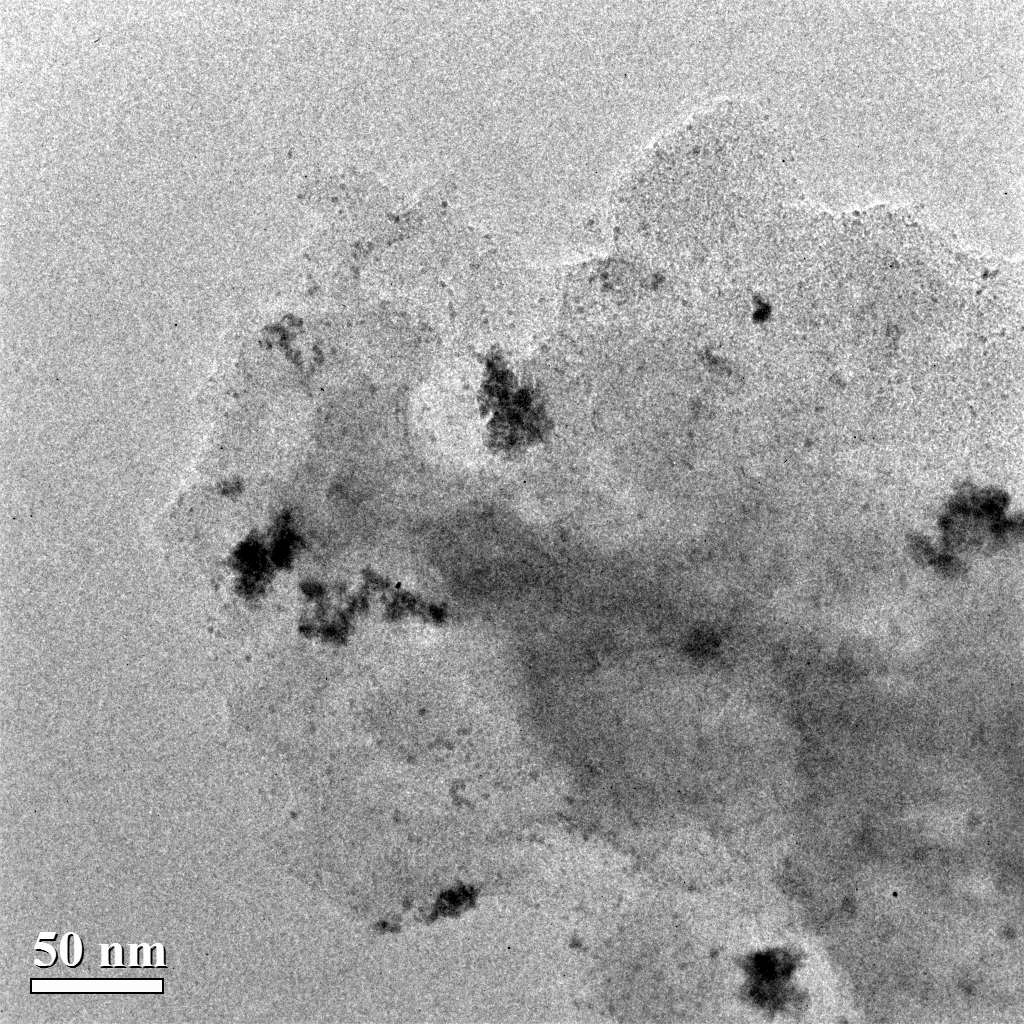

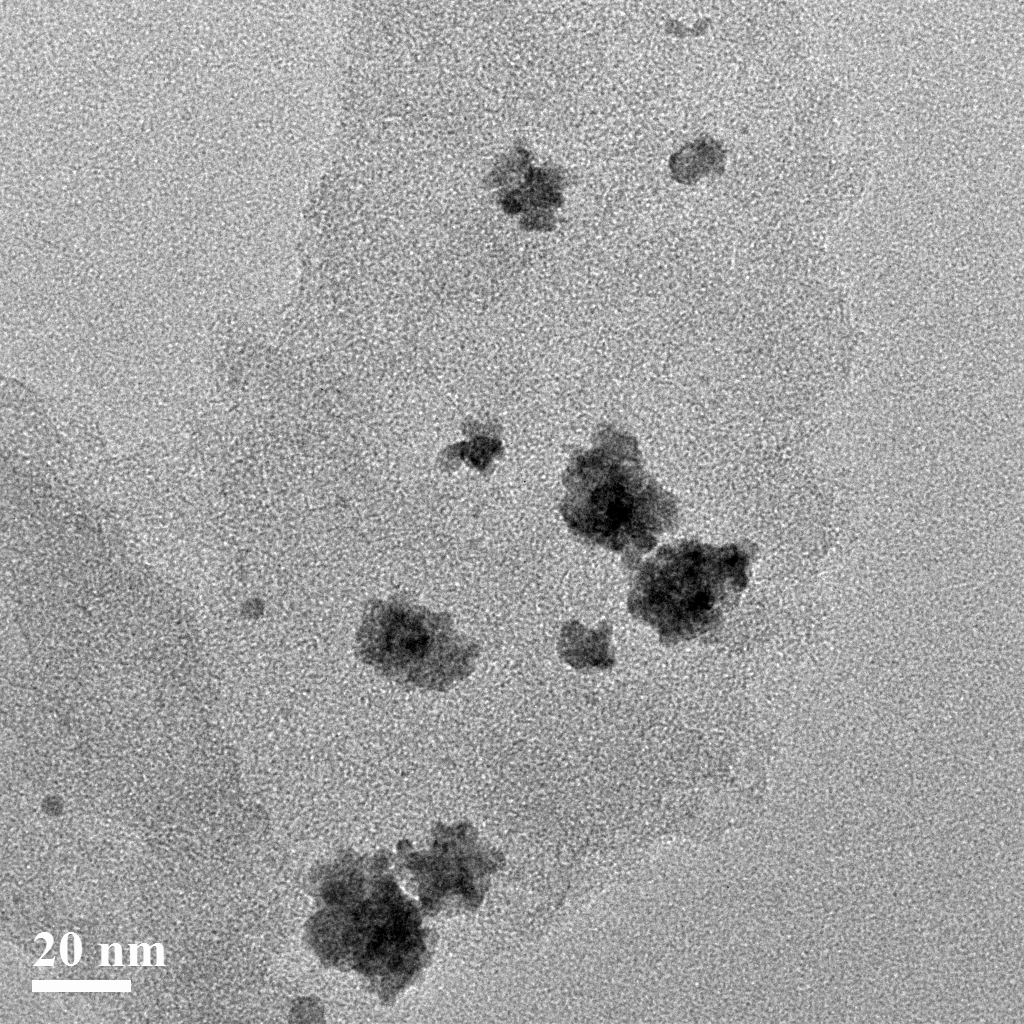


**(a)**

**(b)**

**Fig. S6** TEM images of Pt_1_Ru_1_ catalyst (a) and Pt_0.5_Ru_2.5_ catalyst (b).


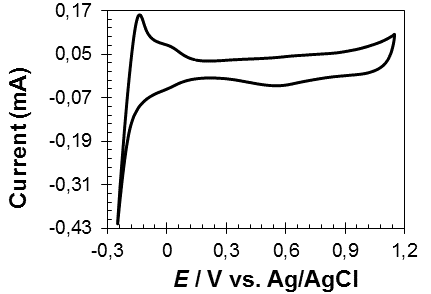

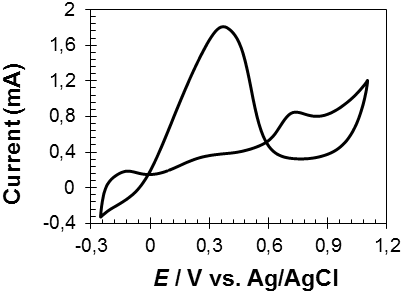

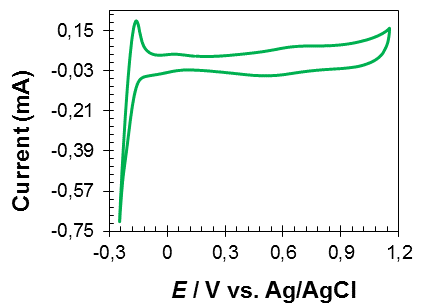

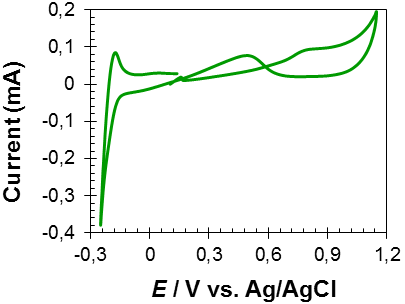

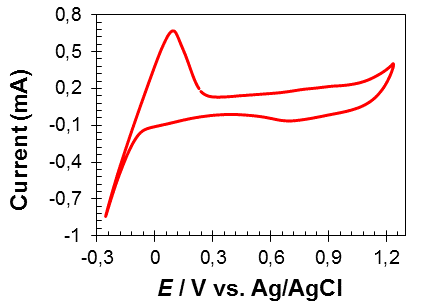

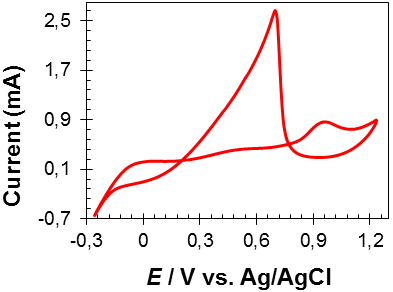

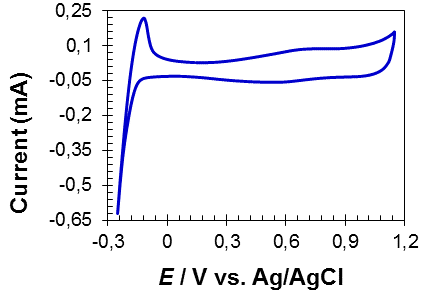

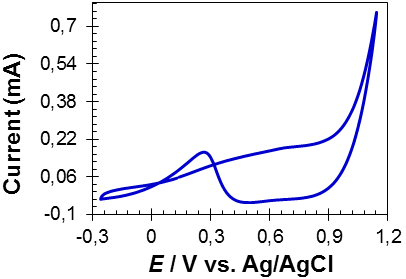


**(a)**

**(b)**

**(c)**

**(d)**

**Fig. S7** CV curves of PtRu catalysts with atomic ratios 2:1 (a), 1:1 (b), 1:2 (c) and 0.5:2.5 (d) run in the absence (left-side) and presence of 1 mol L^–1^ HCOOH (right-side).


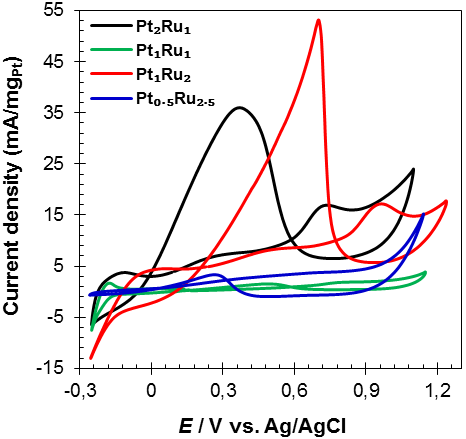

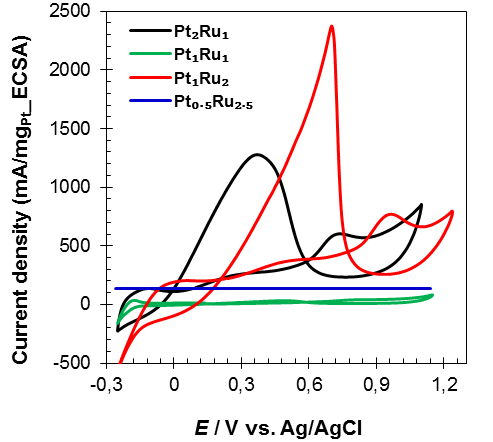


**(a)**

**(b)**

**Fig. S8** CV curves of PtRu catalysts normalized with Pt loading (a) and with ECSA values (b).

**Fig. S9** CV curves of MPC supported PtRu catalysts run in in H_2_SO_4_ (0.5 mol L^–1^) and HCOOH (1 mol L^–1^) mix-solution.

**Fig. S10** Nyquist plot of MPC 950 supported catalyst in H_2_SO_4_ (0.5 mol L^–1^) and HCOOH (1 mol L^–1^) solution.
